# Role of internet use, mobile phone, media exposure and domestic migration on reproductive health service use in Bangladeshi married adolescents and young women

**DOI:** 10.1371/journal.pgph.0002518

**Published:** 2024-03-04

**Authors:** Anita Pickard, Md Irteja Islam, Md Sabbir Ahmed, Alexandra Martiniuk

**Affiliations:** 1 Faculty of Medicine and Health, School of Public Health, The University of Sydney, Sydney, New South Wales, Australia; 2 Research, Innovation and Grants, Spreeha Foundation, Gulshan 2, Dhaka, Bangladesh; 3 Centre for Health Research, The University of Southern Queensland, Toowoomba, Queensland (QLD), Australia; 4 Department of Development Studies, Daffodil International University, Savar, Dhaka, Bangladesh; 5 Department of Community Health and Epidemiology, College of Medicine, University of Saskatchewan, Saskatoon, Canada; 6 Office of the Chief Scientist, The George Institute for Global Health, Newtown, New South Wales, Australia; 7 Dalla Lana School of Public Health, The University of Toronto, Toronto, Ontario, Canada; African Population and Health Research Center, KENYA

## Abstract

Numerous studies have identified factors that are associated with increased access to reproductive health services in lower-middle-income countries (LMICs). However, limited studies examined the influence of access to internet or a mobile phone, media exposure and domestic migration on reproductive health services use in LMICs like Bangladesh. This study investigated the role of such factors on the use of contraceptives, antenatal care (ANC) and postnatal care (PNC) by married adolescents and young women in Bangladesh and whether it was varied by area. Secondary data for 1665 married women aged 15–24 years, sourced from the 2019 Multiple Indicator Cluster Surveys, were included in both bivariate analyses and logistic regression modelling to examine the role of access to internet and/or mobile phone, media exposure and domestic migration on the outcome variables (contraceptive, ANC and PNC). All regression models were controlled for age, wealth, education and number of existing children. Among all participants, 69.8% were aged 20–24 years and 85.6% lived in rural areas. Of the total sample, 67.5% used contraceptives, 75.7% utilised ANC and 48.7% accessed PNC. Domestic migration significantly increased contraceptive use, with women who had moved locally within the last five years 1.84 times more likely to use contraception than those who had never moved (95% CI: 1.41–2.41, p<0.001). Women with internet or mobile phone access were more likely to receive ANC (aOR: 1.57, 95% CI: 1.22–2.00, p<0.001) compared to those without internet/mobile phone access. Media exposure was found to increase the likelihood of receiving ANC in urban areas. No significant influence was found on the use of PNC. Internet/mobile-based platforms are promising avenues for public health messaging regarding ANC in Bangladeshi married adolescents and young women. Further research is required into determinants of PNC service use in low-resource settings.

## Introduction

Access to quality reproductive healthcare services is considered a fundamental sexual and reproductive health right and is known to improve health and economic outcomes for mothers and children [1–4]. Quality reproductive healthcare is key to achieving sustainable development goal (SDG) 3: Ensure healthy lives and promote well-being for all at all ages, through reduction of maternal and infant mortality and ensuring access to family planning. It is also important for SDG 5: Achieve gender equality and empower all women and girls [5]. It is estimated that 290,000 pregnancy-related deaths occur each year in developing nations due to inadequacies in maternal healthcare, and there are 1.1 billion women of reproductive age in need of family planning services [1,6]. Quality reproductive healthcare services during ante- and immediate postnatal periods are particularly important for adolescents who have an increased risk of hypertension (pre-eclampsia), anaemia and preterm labour leading to preterm delivery [1,7,8].

In 2019, the highest number of women with an unmet need for modern contraception, 87 million, resided in Southern Asia [4,9]. At 64%, the contraceptive prevalence rate in Bangladesh is both higher than the Southern Asian average (52%), the lower middle-income country average (51%) and the surrounding country rates [10,11]. However, 10% of married women and 12.7% of married adolescents aged 15–19 years in Bangladesh have an unmet need for contraception [10,12]. It must also be mentioned that data on unmet needs for family planning services only exist for married adolescents, missing a large number of unmarried young women who also need access to such services [12]. Bangladesh’s 4^th^ Health, Population and Nutrition Sector Program aims to increase the contraceptive prevalence rate to 75% by 2023 and reduce discontinuation from 30 to 20% [13]. Moreover, the number of women in Bangladesh receiving four or more antenatal care (ANC) visits has declined in recent years and at 41%, is lower than the surrounding countries such as India, Pakistan, Myanmar and Nepal [10,14]. It should also be noted that the World Health Organisation (WHO) now recommends eight or more ANC visits and the lowest attendance rates are seen in Southern Asia at 49% [15]. Immediate postnatal care (PNC) continues to be one of the most neglected aspects of maternal and child health care despite most maternal and infant deaths occurring during this time [16–18]. The percentage of women in Bangladesh who received an immediate PNC check from a medically trained professional in the two days following birth was 55% in 2022 [10]. The Bangladesh National Strategy for Maternal Health 2019–2030 aims for 50% of women to receive four or more ANC visits and PNC from a medically trained provider by 2020, with this goal increasing to 80% by 2025 and 100% by 2030 [19]. Bangladesh is currently not on track for its reproductive health goals, and an increased understanding of determinants of reproductive health service use is necessary, particularly for at-risk groups such as young women and adolescents.

Previous literature shows that in Bangladesh, living in an urban area, greater wealth and higher education are positively associated with the use of contraceptives, ANC and PNC visits [20–32]. Residing in an urban area is thought to contribute in increased use of reproductive health services in Bangladesh and internationally due to increased health service coverage and wealth in these areas [21,29]. Further, wealth has been not only associated with a better ability to pay for care, but with increased involvement in decision-making and female empowerment, which itself is positively associated with the use of reproductive health services [21,33–40]. Wealthier families often achieve higher education and a woman’s education as well as that of her husband has been positively associated with reproductive health service use in Bangladesh and other LMICs [21,22,24,28,29,41–47]. Older women in Bangladesh generally have greater use of contraceptives and ANC, however, the association between age and PNC use is still unclear [21,28,31,37,38,48]. Contraceptive use also increases with the number of existing children a woman has [33,44,49–51]. Therefore, age, area of residence, education, wealth and number of existing children have been included in this study as covariates.

Prior evidence, including studies from other LMICs, suggest internet access can increase contraceptive use and domestic migration influences the use of ANC services [52–55]. However, these influences have not been investigated in Bangladesh. Additionally, previous research from Bangladesh suggests media plays a role in contraceptive use and ANC but there has been no research in Bangladesh of the role of media on obtaining PNC [36,56].

A previous study involving college students in Bangladesh found that use of mobile phones to access sexual and reproductive health information showed potential for bypassing traditional knowledge gatekeepers, to enable students to make their own decisions around reproduction, particularly for young women [57]. However, this influence of information via mobile phones was largely still dependant on the contributions of family, elders, and health service providers, in particular pertaining to what information was ‘appropriate to know’ and what information should be shared with peers. A further study which evaluated using telehealth for ANC and PNC in Bangladesh during the COVID-19 pandemic showed promise but concerns remained around access to technology and the nature of some care procedures that could only be provided in person [58]. There is a need to understand the current influence of internet and/or mobile phones on reproductive health service use in Bangladesh. Importantly because knowing more about this is likely to support the implementation of potentially efficient and effective initiatives to provide or support the delivery of ANC and PNC. It is highly likely, given the experience of other countries globally, that the internet and mobile phone will be an ever growing conduit of sexual and reproductive health information in Bangladesh.

Globally, rapid urbanisation sees domestic migration from rural to urban areas increasing and was predicted to cause a 93% increase in the urban population between 2000 and 2020 in Bangladesh [59]. Evidence suggests domestic migrants are more likely to use contraception and ANC, possibly due to increased targeting of health information to newer community members [60], and that urban-rural return migration sees an increase in positive attitudes towards family planning and increased knowledge of self-controllable contraceptives from women returning to the countryside [52,53]. A recent study in Nepal found that urban-to-urban migrant women and urban non-migrant women were more likely to use contraceptives than those who migrated from rural-to-rural areas. Moreover, the study also noticed that urban-to-urban migrant women and rural-to-urban migrant women were more likely to receive four ANC visits than those who migrated between rural-to-rural areas [61]. However, this has not been thoroughly investigated in Bangladesh. One study showed rural-urban migrants in Bangladesh did not show significant differences to modern contraceptive use in Bangladesh [59], but use of other reproductive health services has not been studied. Considering the economic, social and cultural change that can be caused by domestic migration, it’s role as a potential barrier or facilitator of reproductive health service use should be investigated along with other factors including mobile phone, internet use and/or media exposure.

Further, current influences such as the effects of the COVID-19 pandemic are yet to be fully understood, however, an initial review of COVID-19 impacts on sexual and reproductive health services in Bangladesh found that family planning had reduced by 23%, with a 31% decrease in ANC visits [62,63]. A model of COVID-19 impacts in 118 LMICs suggested postnatal care could reduce by 18–51.9% (45). Considering the potential impact of COVID-19 on service delivery, it is more important than ever to ensure family planning measures are targeted, preventing the reversal or further stagnation of contraceptive trends and ensuring Bangladesh achieves its reproductive health goals. This is particularly important for at-risk groups such as adolescents and young women. This research aims to investigate the influence of internet and mobile phone access, media exposure and domestic migration on the use of contraceptives, ANC and PNC by married adolescents and young women aged between 15–24 years in Bangladesh.

## Methods

### Data source

Data was sourced from the publicly available MICS 2019 conducted by the Bangladesh Bureau of Statistics in collaboration with UNICEF Bangladesh [64]. A two-stage, stratified cluster sampling approach was used in both surveys covering all administrative districts (N = 64). Enumeration areas from the 2011 Bangladesh Population and Housing Census were classified as primary sampling units. The first stage selected primary sampling units using a probability proportional to size sampling procedure based on the number of households reported in the 2011 census for that enumeration area. Households in each primary sampling unit were listed and 20 households were selected using a systematic random sampling process from each. The total sample size for the 2019 MICS was 64, 400 households. The same sample weighting method based on the 2011 census probability based on population size was used. Data was drawn from the *Questionnaire for Individual Women*, translated to Bengali and administered individually, to all women aged 15–49 years living in sampled households. Verbal consent was obtained for each respondent and those aged 15–17 years, parent/guardian/caregivers’ consent was obtained in advance of the child’s assent. Taking all adolescent respondents aged 15–24 years with complete data for the variables analysed, the 2019 total sample was n = 1665. The total MICS sample size was 22166 and data was excluded due to the respondent not being asked about reproductive health or no response was recorded. Data were collected between January and June 2019. Further details of the MICS sample design and data collection methods are described elsewhere [65].

### Outcome variables

Dependent variables were the use of contraceptives, use of antenatal care (ANC) services and use of immediate postnatal care (PNC) services (mother and/or baby). Women were asked what type of contraception they were currently using. The response “not using any contraception” was coded as 0 (no, not using contraception) and responses “modern method”, “traditional method”, “folkloric method” or “both modern and traditional” were coded as 1 (yes, using contraception). The response “prefer not to answer” was treated as missing data and omitted. Women were asked whether they received ANC and the responses were coded as 0 (no) and 1 (yes). To analyse immediate PNC, if neither the mother nor the baby was checked after the delivery was over, the response was coded as 0 (no, did not receive PNC) and if either mother or baby was checked the response was coded as 1 (yes, received PNC).

### Explanatory variables

In this study, we considered three exposure variables, namely–(i) access to the internet or a mobile phone, (ii) media exposure, and (iii) domestic migration. Response for ‘access to the internet or a mobile phone’ was coded as ‘1’ if the women responded ‘yes’ to having access to the internet and/or a computer or access to a mobile phone (regardless of whether it was a smartphone or not) and coded as ‘0’ if the respondent did not have access to either. Exposure to mass media was defined as women who, at least once per week, watch tv, read the newspaper or listen to the radio, and responses were coded as either 1 (yes) or 0 (no). Women were asked how long they had been living in the current location and for analyses we categorised and coded the variables into the following to specify domestic migration: 0 (never moved from current location), 1 (moved within the last five years) and 2 (moved over five years ago). Based on the literature age (15–19 years, 20–24 years), education level (completed primary education or below, completed an education above primary but below secondary, completed secondary education or above), wealth index (first/poorest, second/poor, third/middle class, fourth/rich, fifth/richest), area of residence (urban and rural), and number of children ever born (1 or 2+) were included as covariates. The categorisation of urban and rural areas in both MICS data sets was in line with established definitions used by the Bangladesh Bureau of Statistics for the Population and Housing Census 2011 [65].

### Statistical analysis

Descriptive analyses were used to calculate the frequencies (n) and percentages (%) of outcome variables, and explanatory variables for the total sample. Chi-squared tests were used to investigate the bivariate associations between explanatory and outcome variables. Covariates found to have a significant (p<0.05) influence on the outcome variables were adjusted in multiple logistic regression models. Further, we conducted regression analysis stratified by area. Adjusted odds ratios (aOR) with 95% CI were reported. Data were analysed using the ‘SVY’ set package of the Stata 14.1 version (StataCorp, College Station, TX, USA) to consider the complex survey design. Further, the assumptions of logistic regression models were examined by the application of the Hosmer-Lemeshow goodness-of-fit test.

### Ethics

To gain access to the MICS, our online request and short research objective were approved by the UNICEF MICS team [66]. The survey protocol was approved by the technical committee of the Government of Bangladesh led by the Bangladesh Bureau of Statistics [65]. Written informed consent was obtained from parent/guardian for the study participants (adolescents/young women) aged less than 18 years. All participants were aware that information was voluntary, confidential and anonymous. Participants could refuse to answer any question and cease the interview at any time.

## Results

The characteristics of the sample are displayed in Table 1. The majority of women interviewed were aged 20–24 years (n = 1163, 69.8%) and lived in rural areas (n = 1425, 85.6%). Most belonged to either the poorest or second poorest wealth quintiles (n = 991, 59.5%) and had achieved between a primary and secondary level of education (n = 900, 54.1%). Over two thirds of participants did not have access to the internet or a mobile phone (n = 1121, 67.3%) and over half did not engage with mass media (TV, newspaper, radio) at least once per week (n = 923, 55.4%).

**Table 1:** Demographic characteristics and rates of exposure to media, internet and migration of married women aged 15–24 years in Bangladesh.

Characteristics (Total, n = 1665)		n (%)
Total sample		1665 (100)
Media exposure		
	No	923 (55.4)
	Yes	742 (44.6)
Access to internet or mobile phone		
	No	1121 (67.3)
	Yes	544 (32.7)
Domestic migration		
	Never moved	320 (19.2)
	Moved within last 5 years	793 (47.6)
	Moved over 5 years ago	552 (33.2)
Age		
	15–19 years	502 (30.2)
	20–24 years	1163 (69.8)
Place of residence		
	Rural	1425 (85.6)
	Urban	240 (14.4)
Education		
	Primary and below	586 (35.2)
	Secondary and below	900 (54.1)
	Above secondary	179 (10.8)
Wealth		
	First/Poorest	570 (34.2)
	Second/Poor	421 (25.3)
	Third/Middle Class	315 (18.9)
	Fourth/Rich	231 (13.9)
	Fifth/Richest	128 (7.7)
Children ever born		
	1	926 (55.6)
	2+	739 (44.4)

**Fig 1:**
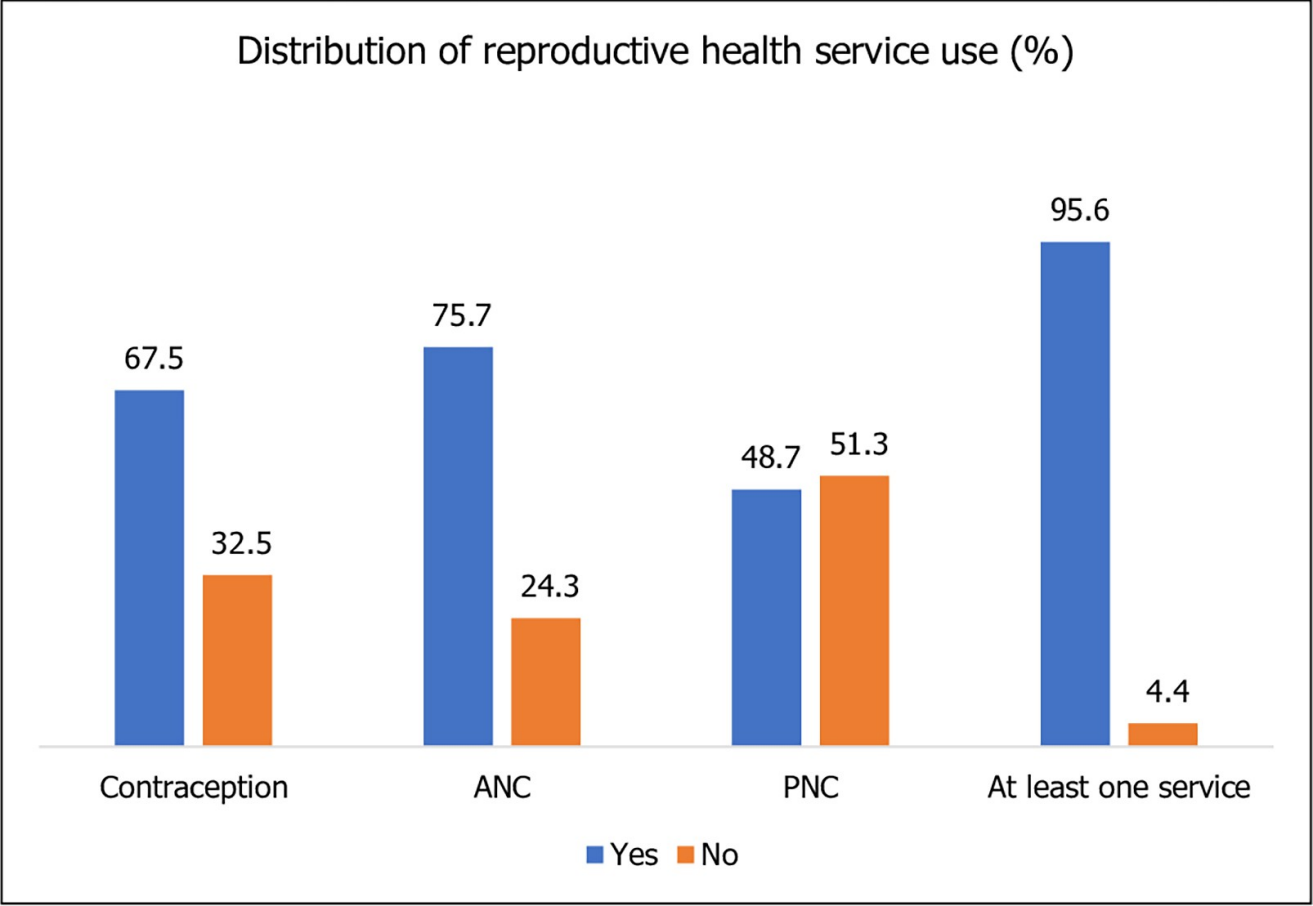
Total sample use of reproductive health services. Descriptive analyses of contraceptive, antenatal and postnatal care use by Bangladeshi married adolescents and young women. The percentage of women using at least one service was also calculated.

Fig 1 shows the distribution of reproductive health service use by all participants. Of married adolescents and young women, 67.5% (n = 1124) used contraceptives in 2019. Antenatal care had the highest proportion of women using the services at 75.7% (n = 1261). Postnatal care was the only service that less than half the women used at 48.7% (n = 811). Almost all women (n = 1592, 95.6%) used at least one service.

The bivariate analysis presented in Table 2 shows history of internal migration (p<0.001) and Wealth (p = 0.050) were significantly positively associated with contraceptive use by the total sample. All independent variables (internet or mobile phone access, exposure to media, and history of internal migration) and covariates (age, education, wealth and the number of existing children) were found to be significantly positively associated with antenatal care of married adolescents and young women in Bangladesh (p<0.010 for all). Only wealth (p = 0.008) was positively associated with use of postnatal care services by the total sample.

**Table 2:** Bivariate analysis of the relationships between sample characteristics and exposures, to use of reproductive health services.

Characteristics		Contraception					Antenatal Care					Postnatal Care				
		Yes		No		p-value	Yes		No		p-value	Yes		No		p-value
		n	%	n	%		n	%	n	%		n	%	n	%	
Media exposure	No	611	54.4	312	57.7		658	52.2	265	65.6		440	54.3	483	56.6	
	Yes	513	45.6	229	42.3		603	47.8	139	34.4		371	45.7	371	43.4	
						0.203					<0.001					0.345
Access to internet and/or mobile phone	No	361	32.1	183	33.8		364	28.9	180	44.6		281	34.6	263	30.8	
	Yes	763	67.9	358	66.2		897	71.1	224	55.4		530	65.4	591	69.2	
						0.486					<0.001					0.094
Domestic migration	Never moved	183	16.3	137	25.3		227	18	93	23		154	19	166	19.4	
	Moved within last 5 years	564	50.2	229	42.3		627	49.7	166	41.1		391	48.2	402	47.1	
	Moved over 5 years ago	377	33.5	175	32.3		407	32.3	145	35.9		266	32.8	286	33.5	
						<0.001					0.007					0.897
Age	15–19 years	335	29.8	167	30.9		402	31.9	100	24.8		243	30	259	30.3	
	20–24 years	789	70.2	374	69.1		859	68.1	304	75.2		568	70	595	69.7	
						0.658					0.007					0.871
Education	Primary and below	380	33.8	206	38.1		387	30.7	199	49.3		277	34.2	309	36.2	
	Secondary and below	625	55.6	275	50.8		721	57.2	179	44.3		440	54.3	460	53.9	
	Above secondary	119	10.6	60	11.1		153	12.1	26	6.4		94	11.6	85	10	
						0.172					<0.001					0.464
Wealth	First/Poorest	376	33.5	194	35.9		373	29.6	197	48.8		265	32.7	305	35.7	
	Second/Poor	308	27.4	113	20.9		319	25.3	102	25.2		221	27.3	200	23.4	
	Third/Middle Class	212	18.9	103	19		260	20.6	55	13.6		142	17.5	173	20.3	
	Fourth/Rich	148	13.2	83	15.3		189	15	42	10.4		105	12.9	126	14.8	
	Fifth/Richest	80	7.1	48	8.9		120	9.5	8	2		78	9.6	50	5.9	
						0.051					<0.001					0.008
Children ever born	1	631	56.1	295	54.5		741	58.8	185	45.8		451	55.6	475	55.6	
	2+	493	43.9	246	45.5		520	41.2	219	54.2		360	44.4	379	44.4	
						0.536					<0.001					0.997

Results from regression modelling for the total sample are shown in Table 3. History of internal migration was found to increase the likelihood of contraceptive use by married adolescent and young women aged 15–24 years. Women who had moved within the last 5 years and who moved over five years ago were respectively 1.84 times (95% CI: 1.41–2.41, p<0.001) and 1.58 times (95% CI: 1.21–2.14, p = 0.001) more likely to use contraception than those who had never moved. Table 3 also demonstrates that women with access to the internet or a mobile phone were 1.54 times (95% CI: 1.22–2.00, p<0.001) more likely to use antenatal care services than those who did not. Women with an education above primary (OR 1.69, 95% CI: 1.32–2.17, p<0.001) and above secondary (OR 1.94, 95% CI: 1.20–3.16, p = 0.007) levels were more likely to use antenatal care services than those with an education below primary level. Women in the wealthiest quintile were over four times as likely to use antenatal care services compared to the poorest (OR 4.2, 95% CI: 1.91–9.23, p<0.001). Married adolescent and young women from all socioeconomic status (from poor to richest) also increased the use of antenatal care services compared to poorest. Having more than two children at the time of the survey reduced the likelihood a woman used antenatal care services compared to those with only one existing child (OR 0.68, 95% CI: 0.50–0.93, p = 0.016). Belonging to the richest wealth quintile was the only factor found to increase the probability of using postnatal care services compared to women in the poorest quintile (OR 1.89, 95% CI: 1.24–2.89, p = 0.008).

**Table 3:** Logistic regression analysis between exposure variables and reproductive health services for married women aged 15–24 years in Bangladesh.

	Contraceptive Use	Received ANC	Received PNC
	aOR (95% CI)	aOR (95% CI)	aOR
Media exposure			
No	Ref.	Ref.	Ref.
Yes	1.19 (0.94–1.50)	1.19 (0.89–1.52)	1.04 (0.84–1.30)
Access to internet or mobile phone			
No	Ref.	Ref.	Ref.
Yes	1.11 (0.89–1.40)	1.54*** (1.22–2.00)	0.79 (0.64–0.98)
Domestic migration			
Never moved	Ref.	Ref.	Ref.
Moved within last 5 years	1.84*** (1.41–2.41)	1.17 (0.85–1.62)	1.01 (0.78–1.32)
Moved over 5 years ago	1.58** (1.21–2.14)	1.38 (0.98–1.95)	0.99 (0.75–1.31)
Age			
15–19 years	-	Ref.	-
20–24 years		0.76 (0.56–1.02)	
Education			
Primary and below	-	Ref.	-
Secondary and below		1.64*** (1.32–2.17)	
Above secondary		1.83** (1.20–3.16)	
Wealth			
First/Poorest	Ref.	Ref.	Ref.
Second/Poor	1.25 (0.93–1.67)	1.36* (1.01–1.85)	1.27 (0.99–1.64)
Third/Middle Class	0.93 (0.68–1.27)	1.81** (1.23–2.56)	0.97 (0.70–1.23)
Fourth/Rich	0.76 (0.53–1.08)	1.66* (0.98–2.33)	0.99 (0.66–1.23)
Fifth/Richest	0.69 (0.44–1.07)	5.02*** (2.32–10.85)	1.89** (1.24–2.89)
Children ever born			
1	-	Ref.	-
2+		0.67* (0.50–0.93)	
Model fit statistics			
Hosmer-Lemeshow Goodness-of-fit test (p-value)	5.96 (0.651)	6.72 (0.566)	6.42 (0.600)

Level of significance p<0.001***

p<0.01**

and p<0.05*.

Variable that were found significant in the bivariate analysis (Table 2) were only included in the models.

Table 4 presents results from regression models stratified by area (rural vs urban for each reproductive service included in this study). Among those who are currently residing in a rural area, who had moved within the last five years and those who had moved over five years ago were 1.85 times (95% CI: 1.38–2.47, p<0.001) and 1.49 times (95% CI: 1.09–2.02, p = 0.012) more likely to use contraceptives than those who had never moved, respectively. Of women in urban areas, those who had moved over five years ago were 2.33 times (95% CI: 1.02–5.31, p = <0.05) as likely to use contraceptives than those who had never moved. Further, among women residing in urban areas, who belonged to the second poorest wealth quintile (OR 4.38, 95% CI: 1.50–12.82, p = 0.007) and the wealthiest quintile (OR 2.58, 95% CI: 1.09–6.07, p = 0.03) were more likely to use contraceptives than the poorest urban women. Table 4. also demonstrates that both rural (OR 1.48, 95% CI: 1.14–1.92, p = 0.003) and urban (OR 2.77, 95% CI: 1.29–5.93, p = 0.009) women were more likely to use antenatal care services if they had access to the internet or a mobile phone compared to those in the same area who did not. Women in urban areas were almost three times as likely to use antenatal care services if exposed to media at least once per week (OR 2.78, 95% CI: 1.13–5.93, p = 0.08) compared to those who did not have the exposure. Urban women with two or more children were 0.31 times less likely to use antenatal care services than urban women with one child (OR 0.31, 95% CI: 0.14–0.67, p = 0.003). Higher education and wealth were found to increase the likelihood of rural women using antenatal care services compared to their respective counterparts. No factors were found to be significantly associated with the increased use of postnatal care services in stratified analysis by area.

**Table 4:** Logistic regression analysis between exposure variable and reproductive health service use, stratified by area, for married women aged 15–24 years living in rural (n = 1425) and urban (n = 240) areas in Bangladesh.

	Used contraceptives		Received ANC		Received PNC	
	Rural	Urban	Rural	Urban	Rural	Urban
	aOR (95% CI)	aOR (95% CI)	aOR (95% CI)	aOR (95% CI)	aOR (95% CI)	aOR (95% CI)
Media exposure						
No	Ref.	Ref.	Ref.	Ref.	Ref.	Ref.
Yes	1.21 (0.94–1.56)	0.87 (0.45–1.71)	1.04 (0.79–1.39)	2.78** (1.31–5.93)	1.02 (0.81–1.29)	1.10 (0.60–2.03)
Access to internet and/or mobile phone						
No	Ref.	Ref.	Ref.	Ref.	Ref.	Ref.
Yes	1.12 (0.87–1.43)	1.36 (0.71–2.61)	1.48** (1.14–1.92)	2.77** (1.29–5.93)	0.85 (0.67–1.07)	0.49 (0.26–0.91)
History of internal migration						
Never moved	Ref.		Ref.	Ref.	Ref.	Ref.
Moved within last 5 years	1.85*** (1.38–2.47)	1.72 (0.79–3.73)	1.28 (0.91–1.80)	0.61 (0.19–1.92)	0.95 (0.72–1.27)	1.60 (0.76–3.38)
Moved over 5 years ago	1.49* (1.09–2.02)	2.33* (1.02–5.31)	1.30 (0.90–1.88)	1.71 (0.53–5.49)	0.92 (0.68–1.25)	1.62 (0.75–3.51)
Age						
15–19 years	-	-	Ref.	Ref.	-	-
20–24 years			0.76 (0.56–1.04)	0.79 (0.26–2.43)		
Education						
Primary and below	-	-	Ref.	Ref.	-	-
Secondary and below			1.73*** (1.33–2.25)	1.78 (0.74–4.27)		
Above secondary			2.05** (1.22–3.44)	1.01 (0.21–4.69)		
Wealth						
First/Poorest	Ref.	Ref.	Ref.	Ref.	Ref.	Ref.
Second/Poor	1.30 (0.97–1.73)	2.32 (0.08–6.72)	1.42* (1.04–1.94)	0.99 (0.25–3.93)	1.32 (1.01–1.72)	1.20 (0.48–3.61)
Third/Middle Class	0.88 (0.65–1.20)	4.38** (1.50–12.82)	1.75* (1.19–2.56)	2.62 (0.53–12.74)	1.01 (0.75–0.37)	0.57 (0.23–1.42)
Fourth/Rich	0.79 (0.55–1.15)	1.62 (0.72–3.64)	1.69* (1.04–2.75)	0.86 (0.26–2.81)	0.99 (0.69–1.42)	0.79 (0.35–1.79)
Fifth/Richest	0.43* (0.25–0.73)	2.58* (1.09–6.07)	10.66** (2.52–45.18)	1.21 (0.29–4.91)	1.40 (0.82–2.38)	1.91 (0.93–5.16)
Children ever born						
1	-	-	Ref.	Ref.	-	-
2+			0.78 (0.56–1.09)	0.31** (0.14–0.67)		
Model fit statistics						
Hosmer-Lemeshow Goodness-of-fit test (p-value)	4.52 (0.807)	2.45 (0.964)	12.08 (0.147)	5.61 (0.690)	5.84 (0.665)	3.52 (0.898)

Level of significance p<0.001***

p<0.01**

and p<0.05*.

Covariates that were found significant in the bivariate analysis (Table 2) were only included in the adjusted models.

### Model fit statistics

All the models in Tables 3 and 4 shows that the p-values of the Hosmer-Lemeshow statistic (Goodness-of-fit test) were not statistically significant (p>0.05), which indicates good logistic regression model fit.

## Discussion

This study investigated the factors that influence reproductive health service use by married women aged 15–24 years in Bangladesh. Overall, access to the internet and/or mobile phones increased use of ANC services regardless of women’s area of residence. Media exposure was found to significantly increase the use of ANC services for women residing in urban areas only. Domestic migration increased contraceptive use, particularly for women residing in rural areas. The influence of sociodemographic factors such as education, wealth, and number of existing children, on the use of contraceptive, antenatal and postnatal care were consistent with previous literature.

Previous studies have found that access to mass media can increase the likelihood of contraceptive and antenatal care service use [27,67], however, this study suggests this finding does not hold for adolescent or young women. One reason for this could be reduced decision-making power of adolescents who may need to rely on partners or in-laws for health-related decision-making compared to older women [68]. In this context, the targeting of family planning, ante- and post-natal care messaging or information may be more influential when addressed to family members of adolescent or young women. Alternatively, the role of technology may have shifted from traditional forms of media (radio, TV, printed news) to online, internet and mobile based platforms for this age group. It is important to note that though access to the internet and/or mobile phones was positively associated with ANC, data regarding how, or how often, the internet or mobiles were used in relation to reproductive health care or information was not captured. A recent review on sexual and reproductive health rights among Bangladeshi adolescents found that the internet is now the second most used form of mass media (still behind TV) and is a promising platform for sexual health messaging [12].

Migration can cause an increased financial burden and stress on families and couples [69–71]. This expenditure and instability may contribute to the increased use of contraception by those who have recently moved found in this study, as these couples or families may not be ready for a child. Internal migration is often also for job opportunities and women who are employed are more likely to use contraception due to the perceived effects of childbearing on career aspirations [24]. The influence of internal migration was more pronounced in this study for women now living in rural areas. International evidence suggests urban-rural return migration sees an increase in positive attitudes towards family planning and increased knowledge of self-controllable contraceptives from women returning to the countryside and may be present in this study [52,53]. Alternatively, women who have migrated due to marriage between rural areas may be more likely to be using contraceptives before the couple decides to start a family.

## Limitations

This study conducted secondary data analysis using pre-COVID-19 data, preventing analysis of COVID-19 impacts on reproductive health services use in Bangladesh. However, it is a valuable analysis of pre-pandemic data and can serve as baseline to observe changes, if any, due to the pandemic on use of reproductive services. These data will assist in understanding future actions required to address any declines in health services due to the COVID-19 pandemic and may help improve the efficiency and cost-effectiveness of future communication and services delivery. As a cross sectional study, this research is unable to determine causal factors of reproductive health service use and only investigate influences. Some bias may also be present in survey responses due to cultural expectations surrounding reproductive health services and childbirth.

## Conclusion

Access to the internet or a mobile phone increases the likelihood of using ANC services and suggests online-based platforms are promising avenues for increasing use of reproductive services. Furthermore, domestic migration is associated with increased access to reproductive health services (contraceptives and ANC) in Bangladesh. More research is required into the determinants of PNC service use by adolescent and young mothers in Bangladesh. This work highlights the changing influences on the use of reproductive health services in Bangladesh as the country experiences rapid economic growth, urbanisation and increased use of technology within the general population. This study can direct further research and advise policy planning to address women’s knowledge regarding sexual and reproductive health services as well as potentially improve women’s access to reproductive and maternal healthcare in Bangladesh.
